# Historical Sketch of the Progress of Medicine in the Year 1809

**Published:** 1810-07

**Authors:** 


### Medical and Physical Journal.

VOL. XXIV.] JULY, 1810. [NO. 137.

*Printed for R. PHILLIPS, by W. Thorne, Red Lion Court, Fleet Street, London.*

### HISTORICAL SKETCH OF THE PROGRESS OF MEDICINE IN THE YEAR 1809. BY MR. ROYSTON.

### No. 4.

( To be continued Annually. )

" *Rien n'est Beau, que le Vrai.*"——VOLTAIRE.

" *The pleasures of sudden wonders are soon exhausted, and the mind can only repose on the stability of truth.*"——JOHNSON.

THOUGH often enchanted by the corruscations that flash from the fire of imagination, or subdued by the logical subtleties of scholastic disquisition, we have especially to lament that the exercise of the mental principle has so seldom been governed by an adherence to truth. The progress of science has too commonly been marked by a delusive colouring given to facts; by the ingenuity of hypothesis retarding discovery, and by the influence of theory repressing the advances to knowledge. The art of medicine, possibly, more than any other, has suffered by a disposition to turn opinions into principles; and with principles thus manufactured, to erect labored fabrics, and dignify the elaborate visions with the denomination of system. The late ingenious and lamented Dr. Currie of Liverpool, who was himself an instance how far an acquaintance with Nature might be carried by cautious induction, observed, that " the great obstruction which men have in all ages experienced in the pursuit of knowledge, has arisen from the promptitude in the human mind to decide in regard to causes. To the weak and the ignorant, presumption is as natural as doubt is intolerable, and with such, belief is almost always a creation of the imagination. Nor do these observations apply to weakness and ignorance only: to retain the mind unprejudiced and undecided, in the investigation of striking and interesting phænomena, till, by the painful steps of induction, their hidden cause is revealed, is an effort of the most difficult kind, and requires the rarest and highest powers of the understanding. The records of every part of science bear ample testimony to this truth, particularly the records of medicine. The most eminent physicians in every period of the world, impatient of observing and delineating, have been eager to explain, and even to systematize; and the science of life owes its corruptions more to the misapplication of learning, than even to the dreams of superstition."

The fall of the systems of Galen and Boerhaave was not effected by the mutability of fashions:—their component principles wanted the foundation of truth. Hypothesis, however ingenious, is not formed to last: ascertained facts alone are durable. The present age is, perhaps, more perfectly sensible of this, than any preceding. If the period for systematizing has not entirely passed away, there is a greater desire to collect facts, than has been manifested at any former time: and it frequently occurs, that the plain narrative obtains an attention that once was given only to the subtle theorist, and the fabricator of plausible hypotheses. While it is, however, matter of gratulation, that Brown and Darwin have no successors in the science of system-building; we cannot look with indifference, nor without apprehension, on the spirit that impels to the multiplication of books. The art, perhaps, peculiar to modern times, of manufacturing literary wares, is extending with incredible rapidity into every branch of human knowledge. Books are easily formed out of books; and the scissars is the magical instrument that promulgates science and philosophy. When the plagiarism is not thus direct, and the pen has actually been employed, a flippant flourish of style, splendor of paper, neatness of type, and elegance of decoration, render superfluous the labors of investigation and the toil of research. Medical literature has not escaped from this delusion of the age. When we look on the fearful multitude of medical quartos, octavos, and duodecimos, that have issued from the British press in the present year, can it be doubted to what extent this modern art has been exercised? Even the labor of describing this multitude, becomes a subject of alarming contemplation. Upwards of seventy new works, and thirty new editions, on the subject of medicine, have in this period been published in London. Of this number, how many have enlightened the science of medicine, how many have added improvement improvement to the practice, or have more clearly explained what was yet imperfectly known; the writer, whose aim is not taken from the masked battery of anonymous criticism, would be imprudently bold to declare. Without daring to alarm this medical hive of the *genus irritabile,* by individual applications, it may be truly asserted, that improvement in the science of medicine has not kept pace with the extension of its literature.

In traversing the circle of medical science, various branches of the art will appear to have different degrees of value. A judicious employment of the practical part, or that, the direct object of which is the cure of disease, it must be admitted, is of the most importance. But the power of properly employing this part, cannot be acquired but by a patient attention to preliminary and auxiliary studies. Of these we have been accustomed to consider Anatomy and Physiology as of primary efficacy. No cause has arisen that should induce a change in this opinion; but it is to be regretted, that the present year affords so few materials. *Gall* and *Spurzheim Recherches sur le systéme nervaux en general, et sur celui du cerveau en particuliere.*—*Watts's* anatomico-chirurgical views of the nose, larynx and fauces; with *Pettigrew's* views of the basis of the brain and cranium, are the principal separate anatomical productions of 1809. What has been produced on the continent, beside the work of Gall and Spurzheim, the precarious and scanty intelligence, which by stealth only arrives, does not enable us to state.

Few of our readers but will recollect the popular reputation of Dr. Gall, and the controversies that have existed upon his novel and visionary system respecting the external appearances of the cranium, as indicatory of the propensities, powers, and capacities of human intellect. The wild enthusiasm that appeared to direct the opinions of this philosophizing anatomist, convinced us that his hypothesis was framed of fragile materials, and that a scanty period would terminate its progress. This opinion was not founded, however, on any direct publication of Gall, on the subject of what he quaintly called *Craniology;* but rather on the statements of his pupils and partizans: for, in this case, as often before has occurred, the policy of the leader of the sect, taught him either to disdain, or to fear committing himself, otherwise than in his public lectures.

But subsequent to this, Dr. Gall has submitted his asserted discoveries on the anatomy of the brain, to the physical class of the French National Institute, in a memoir, moir, entitled "Inquiries into the Nervous System in general, and the Brain in particular." This body appointed a committee, consisting of M. M. Tenon, Portal, Sabatier, Pinel, and Cuvier, to examine this Memoir. The report of these commissioners bears the date of April 1808, and is as clear and perspicuous an explication of the doctrines of Gall and Spurzheim, as the obscure and intricate nature of the subject of this Memoir admitted. It is evident from this report, that the investigations of these anatomists were always ingenious, sometimes highly satisfactory; and that Messrs. Tenon, &c. frequently agreed with them, though they were sometimes compelled to dissent. From a production, in itself highly ingenious, and employed on a subject of such decided interest as the development of the structure of a part of the human frame, from whence is believed to spring the susceptibility to impression in the organs of sense, and the distribution of feeling over the whole system, and which has also a direct connexion with the mental operations, it would be extremely satisfactory to quote largely, did the space allotted to this Sketch admit. We must be content, however, to refer to the Report itself, or to a translation of it in the Edinburgh Medical Journal, January 1809. It appears that Messrs. Gall and Spurzheim were not satisfied with this Report of the Commissioners, who they will scarcely allow, notwithstanding their frequent intercourse and repeatedly dissecting the brain with them, to have understood the subject. This opinion is fully expressed in their observations *sur la Rapport, &c.* These *Recherches* will probably raise the character of *Gall* in this country; and he may no longer be known only as a philosophical charlatan, but rather as an anatomist who patiently inquires into the structure of the nervous system; and often as a physiologist of happy conjecture on the functions and operation of the brain.

From these abstruse and bewildering speculations, which leave the jaded intellect uncertain of what is, or is not, to be believed; it is satisfactory to turn to productions where something like common sense and demonstrable fact appear. Of this kind is the work jointly produced by Mr. Watt and Mr. Lawrence. To correct views of Nature in this, are joined descriptions of the parts delineated, so perspicuous that they leave but little farther to be wished on the subject. The anatomico-chirurgical views of the nose, mouth, larynx, and fauces, present explanations of parts not readily comprehended by the student from actual dissection; but who will, with the assistance of these plates, soon soon overcome this difficulty. The "Views of the basis of the brain and cranium, with a dissertation on the origin of the nerves," by Mr. Pettigrew, is to be considered as the work of a young man, ardent in the pursuit of professional knowledge. We are concerned that in this eagerness to signalize himself he has become obnoxious to the castigation of a harsh critic, who accuses him (Ann. Med. Rev. & Register, v. ii. p. 320.) of unpardonable faults in composition, and of bungling piracy. Without designing also to become accusers, we cannot but remark, that Mr. Pettigrew claims the discovery of certain rules for ascertaining the situation of the trunks of cutaneous nerves, *which rules have been publickly taught by Mr. Brookes, for many years, both in his Dissecting-room, and at his Lecture.* And not only have these rules been applied by Mr. Brookes to the *par trigeminum;* but he has likewise been in the constant habit of pointing out methods for discovering the exact seat of the trunks of all the cutaneous nerves in the superior and inferior extremities; so that a surgeon, acquainted with what Mr. Brookes has, on this subject, been teaching his pupils for a long time, cannot possibly err, in either dividing these nerves, or exposing them only, as may be intended. We hope Mr. Pettigrew was not aware of these facts, when he says, "*I have endeavoured to lay down an almost invariable rule (*p. 11.*) to accomplish this* (the division of nerves) *to a certainty upon all those ramifications of nerves superficially situated.*"

In addition to the separate anatomical works above mentioned, several ingenious papers have appeared in periodical publications. At the head of these publications may properly be placed the Philosophical Transactions. In the first part of this national work for 1809, Mr. Brodie relates the case of a fœtus, in which the heart was wanting, though it had advanced to considerable fœtal maturity. Its stomach had no cardiac orifice; there was neither colon, omentum, liver, nor gall-bladder; *and the circulation must have been carried on entirely by the arteries.*

Mr. Home details an examination of the vertebræ of the *squalus maximus Linn.* by which he discovered that three pints, at least, of mucous fluid, was found within the intervertebral substance. This apparatus, probably, serves to facilitate the motions of the spine, by affording a fixed centre, round which the vertebræ may move with steady velocity. A similarity of structure was discovered in the spines of other animals. The intervertebral fluid was, however, generally greater in fish than in quadrupeds; the

 rapid rapid motion of the spine in swimming, requiring in them a higher perfection in this apparatus. Mr. Home had an opportunity of further examining the structure of the *squalus maximus*, by one having been accidentally caught on the coast near Hastings, in the night of the 13th of November 1808. In the pursuit of a subject, which seems to have occupied much of his attention, Mr. Home particularly directed his inquiries to the stomach of this fish. In the 2d part of the Phil. Trans. he relates the result of these inquiries. From the examination of this organ, in which was found an immense quantity of pebbles, it is presumed that this species of *squalus* affords the connecting link in the gradation of animal life, between the whale and the cartilaginous fishes. Upon tracing the 6th ventricle of the brain, which corresponds with the 4th in the human subject, to its apparent termination, the *calamus scriptorius*, Mr. Sewell perceived the appearance of a canal, continuing by a direct course into the centre of the spinal marrow. By sections of the spinal marrow, he relates in a letter to the Roy. Soc. it was ascertained that this canal terminated insensibly in the *cauda equina*. As all reasonings on natural subjects should be founded on absolute fact, and all conclusions formed from fair analogies, resulting from such facts, though we may not be prepared now to say what is the design of placing such a canal in the centre of the spinal marrow, we have a satisfaction in recording an appearance that may hereafter explain some law in the yet hidden operations of the brain and nerves.

The first volume of the Transactions of the Medical and Chirurgical Society of London, contains three papers on anatomical subjects. The first of these is a description of two muscles surrounding the membranous part of the urethra, by Mr. Wilson, of Windmill-street. These muscles have the power of diminishing the capacity of the urethral canal. Some practical circumstances respecting the state of the urethra, are explained from the action of these muscles, which have the power, not only of diminishing, but closing up that part of the canal where the membranous portion of the urethra joins the penis. On this Mr. Wilson remarks, that " knowing such muscles exist, we shall not hastily infer, that a permanent stricture of the urethra is formed, because we cannot introduce a bougie or catheter; we shall therefore avoid using those remedies, which, though adapted to the cure of a stricture attended with a morbid alteration of the urinary passage, would, in a case arising from contraction only of these muscles, muscles, prove injurious to the patient. *In cases of retention of urine, where no instrument will enter the bladder, we shall be induced to persevere in the means best adapted to overcome a temporary, but forcible contraction of the muscles. When this has been done, our second attempt to draw off the urine will probably succeed.*" This circumstance, perhaps, explains an occurrence that has been as flattering to one operator as humiliating to another. A surgeon of notorious dexterity has been foiled in his attempt to pass a catheter into the bladder. After long persevering he gives up the point; when another, whose skill he has held in great contempt, passes the instrument immediately, and with apparent ease. This happens from the first attempts having fatigued the muscles, which Mr. Wilson describes, nearly to the exhaustion of their contractile power. In the interval between the exhaustion of the muscular power of the uterus by one labor pain, and its recovery to produce another, the hand is easily passed into its cavity. It is a known fact that, in dislocations of the humerus, long continued efforts exhaust the muscles which resist the return of the bone to its place, and render its reduction easy. The being liable to exhaustion of power by continued effort seems a general law of the muscular fibre, and may frequently be applied to practical purposes. Mr. Wilson's paper is accompanied by a drawing, which gives a distinct view of these muscles, with the relative situation of the neighbouring parts.

Connected with anatomy are two more papers in this volume. One of these, is the case of a boy who had a fœtus found in his abdomen. Mr Young, the relator of this curious fact, states, that the boy, who is the subject of it, was born in May 1807, and died in February 1808. In his short life he suffered much from disease, the origin of which seemed to be among the abdominal viscera. On opening the body, a substance was found in a cyst within the abdomen, having unequivocally the shape and character of a human fœtus. A minute account is given of the state of the abdominal viscera in general, accompanied with plates, which render the history very intelligible. A similar phenomenon occurred at Paris a few years since, and was published in a *bulletin de l'Ecole de Medicine.* The other paper contains an historical account of Philip Haworth, a boy, in whom the signs of puberty commenced at the early age of one year. A minute history is given of this boy by Mr. White, accompanied with notices of similar deviations from the course of nature. The subject of this paper is

 still still living, and has now entered his fifth year. When we saw him, some time in 1809, he was extremely robust, and had, with perfect marks of virility, the intellect of a child of three years old.

Physiology, a branch of science certainly more fascinating, though less correctly understood than Anatomy, has, during this year, experienced considerable acquisitions. Dr. Reeve, whom we had an opportunity to notice in the Report for 1808, as the author of an ingenious history of Cretinism, has published an Essay on the Torpidity of Animals. Among the phenomena of animal life, the property, or the habit of having suspended some of its most striking functions; and of remaining, for a great length of time, in a state of apparent sleep, without motion, and without nutriment; seems the most extraordinary. On this curious subject, Dr. Reeve has collected a number of facts of considerable interest, and collated the opinions of many celebrated authors, forming an Essay which will both excite to inquiry and assist the inquirer. As this is, we think, [illegible] separate publication expressly on this peculiar state of animal life, in the English language, it has the merit of leading to disquisitions on a curious physiological fact. The property of having the obvious actions of life suspended for a great length of time, without the absolute destruction or separation of the vital principle, is not confined to animals; it is plainly seen among vegetables, for the reduced temperature of every winter occasions it; and by a certain degree of cold, artificially employed, this hybernation of trees has been much extended. At Moscow, M. Demidow, placed apple and pear trees, that came from France, in an ice cellar, and suspended their vegetation for nineteen months: yet these trees, when afterwards subjected to the stimulus of heat, produced flowers and fruit. In some animals, especially of the class *Vermes* and order *Testacea* of Linneus, torpidity was continued a great length of time without destroying life. This has been remarked particularly to happen to the *Helix hortensis*, which has continued in this state for many years, without parting with the vital principle. The leading facts relative to hybernation are arranged under the following heads.

1st. On the approach of winter, many animals retire from exposure to the atmosphere into retreats, where they roll themselves up, and yield to a kind of sleep, which usually continues till the return of warm weather.

In

In this state it is observed, that their temperature is greatly diminished, though it still continues somewhat above that of the surrounding medium.

2d. The frequency of pulsation in the heart and arteries is greatly diminished.

3d. Respiration becomes less frequent, or ceases entirely.

4th. The stomach and intestines cease to act.

5th. Sensation and voluntary motion are lessened, or cease.

The Phil. Trans. of this year afford some specimens of ingenious physiological speculation. The Croonian Lecture, by Dr. Young, on the Functions of the Heart and Arteries, takes a prominent station among these. The learned author particularly directs his investigation to the following heads.

1st. What would be the nature of the circulation of the blood, if the whole of the veins and arteries were invariable in their dimensions, like tubes of glass or bone?

2d. In what manner would the pulse be transmitted from the heart through the arteries, if they were merely elastic tubes?

3d. What can, with propriety, be attributed to the muscular coats of the arteries themselves?

4th. Observations on the disturbances of these, which may be supposed to occur in different kinds of inflammations, and of fevers.

In pursuing this inquiry, much curious research, ingenious speculation, and profound mathematical reasoning are employed. The calculations and experiments of Keil, Hales, and Haller, are resorted to for the decision of difficult points; but whether the main object of the paper, which is to prove, that the circulation of the blood through the arteries is but little promoted by the muscular power of those vessels, is established, remains, at least, doubtful. It is a singular fact, that in the volume where Dr. Young labours to establish this principle, a case of monstrosity should be given, where the circulation was carried on without the assistance of the heart, that organ being entirely wanting. The superiority of fact over speculation is pointedly put by this fortuitous circumstance. The plain narrative of Mr. Brodie will, probably, carry conviction much further than the elaborate and able reasoning of Dr. Young. The power of working up the phantoms of imagination with plausible dexterity, is sometimes given in exextraortraordinary profusion ; and speculations on the functions of animal bodies then mislead in a ratio agreeing with their ingenuity. It is not meant to insinuate, however, that this property or power has been exercised in the Phil. Trans. of this year, with any uncommon adroitness, or redundance of dexterity. On the contrary, there seems a disposition in the Royal Society to urge that the foundation of opinions be laid on actual experiment, and on known or ascertainable facts.*

Physiology has been much indebted to modern chemistry, for the illustration of many phenomena in animal life heretofore inexplicable. Especially, have the laws and effects of respiration been unfolded by this science: and a paper on this subject, by Messrs Allen and Pepys, in the Phil. Trans. of this year, will shew the ardour with which, in this view, it is still cultivated.

Under the denomination of Animal Chemistry much has also been done to ascertain the true properties of the materials, whether solid or fluid, of which the animal frame is compounded. Among those who have laboured on this subject with most effect, Dr. Bostock, of Liverpool, must be noticed for his persevering ingenuity. A paper on the gelatine of the blood, in the Transactions of the Medico Chirurgical Society of London, for this year, will manifest the claim this gentleman has to particular attention. Beside the chemical object of this paper, in it will be found a neat epitome of the history of opinions on the nature and properties of the blood. In the Philosophical Transactions, Dr. Pearson's paper on Expectorated Matter; Mr. Brande's Observations on Albumen, and some other animal fluids, with remarks on their analysis by electro-chemical decomposition;—and Mr. Home's Hints on the subject of Animal Secretions, are properly arranged under the class of Animal Chemistry. The first of these is defective in a practical view, as it does not state "*those properties by which expectorated secretion may be distinguished from expectorated pus.*" Every practitioner has felt how desirable it is to have a criterion, by which to distinguish pus from mucous, as regarding discharges from the lungs. If

* Mr. Knight has made some curious observations on the comparative influence of male and female parents on their offspring. These are inserted in the Phil. Trans. of this year, and deserve attention as speculations on an abstruse, but interesting subject.

If Dr. Pearson has discovered such a criterion, the spirit for scientific improvement indicated in the general tenor of his pursuits, will prevent his long withholding it from the public.

THE practical part of Surgery, has in modern times received so much improvement, as scarcely to resemble what it once was. Every year, indeed, adds something either to its reasoning or operative part; and the present is not wanting of this spirit of improvement. Among the publications on Surgery, in 1809, we notice a second volume of Charles Bell's System of Operative Surgery, and his Letters concerning Diseases of the Urethra.—Wardrop's Observations on *Fungus Hæmatodes.* A Dictionary of Practical Surgery, by Mr. Cooper.—Dr. Serney's Treatise on Local Inflammation.—An Essay on the Nature and Treatment of the Malignant Contagious Ulcer, as it generally appears in the British Navy, by James Little, Surgeon, R. N.—Abernethy's Surgical Observations on the Constitutional Origin and Treatment of Local Diseases; and Aneurism.—And, as the latest production on the Surgery of France, that the designed difficulty of communication with that country has permitted us to examine, *M. Richerand's Nosographie Chirurgicale.*

The observations on the *Fungus Hæmatodes* will deservedly excite considerable attention; and the labour of Mr. Wardrop must reflect credit on himself, and benefit science, notwithstanding he may have extended the appellation far beyond what a sober view of a specific disease can justify. We apprehend this alarming complaint must have existed in all periods, and even before there were records of science: but it has been the fortune of Mr. Burns, of Glasgow, in the year 1800; first, distinctly to point it out in his *Dissertations on Inflammation.* Mr. Hey, of Leeds, in 1803, (*Practical Observations in Surgery*) accurately detailed some cases of this disease, and applied to it the term *Fungus Hæmatodes.* In 1804, an analagous affection, under the name of *Medullary Sarcoma,* was described (*Surgical Observations, &c.*) by Mr. Abernethy. It has sometimes been called soft Cancer: and Mr. Pott mentions a similar disease in the calf of the leg, which he considered as arising from a rupture of the posterior tibial artery. Since the attention of the profession was first excited to this complaint by Mr. Burns, the cases of it seem to have increased with great rapidity. This is to be accounted for, however, by the appellation given to it by Mr. Hey,

Hey, being made to comprehend many morbid alterations of structure, which had before gone by other designations. It is most desirable that a disease so destructive should be fully identified: and if it can be considered as a genus comprehending several species, the generic relation of these, as well as their specific differences, should be clearly defined. Opinions on the cause of this malady are greatly at variance. Some have considered it as arising from a morbid change in the structure of the small arteries, similar to that which, in the large ones, disposes to aneurism. Others look upon it as "a living parasitical animal, nourished by the vital fluid of the patient; and capable of absorbing from the adjacent vessels what is effused from its own." The progress of this disease, when it attacks the eye-ball, is described with great accuracy; and constitutes, perhaps, the chief excellence of Mr. Wardrop's Work. "The first appearance of the *Fungus Hæmatodes,* when it attacks the eye, he says, is observable in the posterior chamber. The pupil becomes dilated and immoveable, and, instead of its natural deep black colour, it has a dark amber, and in some cases a greenish hue, giving to the eye very much of that appearance which is observed in the sound eye of the sheep, the cat, or any of the lower animals. As the progress of the disease advances, the colour becomes more remarkable; and it is soon discovered to be produced from a solid substance, which is forming in the bottom of the eye, and gradually approaching towards the cornea. The surface of this substance is generally rugged, and not unlike what may be supposed to arise from a quantity of effused lymph. In some cases, red vessels may be seen running across the opake body; but these are not the vessels which nourish it, but ramifications of the cerebral artery of the retina. When the disease advances further, the form of the eye-ball begins to alter, acquiring an irregular knobbed appearance; at the same time, the sclerotic coat loses its natural pearly white colour, and becomes of a dark blue, or livid hue. The tumour, by its continued growth, finally occupies the whole anterior chamber, and, in some cases, a quantity of purulent matter collects between it and the *cornea.* At last the cornea ulcerates, and a fungous tumour shoots out from the portion of the diseased substance, contiguous to the ulcerated cornea; and in other cases, the tumour pushes itself through the sclerotic coat. The substance of this fungus is very readily torn; and when a portion of it is separated, parated, or if it be slightly scratched, it bleeds profusely. In other cases, the tumour is of a firmer texture; and if, as sometimes happens, instead of coming through the cornea, it burst through the sclerotic coat, it then pushes before it the conjunctiva, and thus derives a mucous covering. When the tumour becomes very large, portions of the most prominent parts begin to lose their vitality, and separate in sloughs, which have a very offensive and fœtid smell, and are accompanied with the discharge of an acrid serum." The opinions of Mr. Abernethy will always have, as they will always deserve, a considerable share of public attention. It would, however, occasion much regret, if these opinions were becoming hypothetical, and were carried to the extreme of deriving almost every chronic disease from the same source. The "Observations on the Constitutional Origin and Treatment of Local Diseases," is in part a reprint of a former work, with some alterations and additions; particularly a paper on Aneurism, with which the volume concludes. The object of this publication is, to lay before the profession the facts which have come under the author's observation, relative to the disorders of the digestive organs, and their connection with and effects on other parts of the frame..

For some years, a species of ulcer called the *ship, or contagious malignant ulcer*, had afflicted the British Navy to an extent that *was* extremely alarming. Mr. Little, in the pamphlet mentioned in the preceding enumeration, has given the pathology and treatment of this ulcer. But, that the cases of this disease are so decreased in number, as to be, comparatively, an object of slight professional consideration, will appear from the following statement of admissions of patients with it for a series of years, into the Royal Hospital at Plymouth. From May to the 31st of December, 1803, were received 87 cases.—In 1804, received 621.—In 1805, 377.—In 1806, 505.—In 1807, 346.—In 1808, 223.—In 1809, 220.—And to the 30th of April, 1810, 58. In 1804, 19 died in the hospital of this ulcer. In 1806, 23 died. In 1808, 5 died. In 1809, none died. In 1810, to the 30th of April, none. The rise, progress, and decline of this disease, presents a curious fact in the history of the Medical Art, and deserves a minute and unprejudiced investigation. Perhaps the following authentic notices may be of use to its future historian.

Some idea may be formed of the ravages occasioned by this this malady, when it is stated that in 1804, six hundred and twenty-one patients labouring under it, were admitted into a single hospital at Plymouth. In 1805, Sir Edward Pellew, commander in chief in India, stated to the Admiralty that, so destructive was the ship-ulcer in his squadron, and so intractable under every kind of medical and chirurgical treatment, that an apprehension was entertained of some of his ships being depopulated. In 1804 and 1805 it had made destructive inroads upon the health of the fleets off Brest and Ferrol; but the greatest fatality occurred in 1806 on board the *Salvador del Mundo,* then a receiving ship at the port of Plymouth. When in 1804 and 1805, Dr. Andrew Baird, Inspector of naval hospitals, to whom the nation is indebted for rescuing her seamen from an enemy, against which no prudence could protect, no courage prevail, visited the fleet off Brest and the squadron at Ferrol, he found in many ships the progress of this disease truly awful. The representation of Sir Edward Pellew, on the state of his squadron, induced the Lords of the Admiralty to call upon Dr. *Baird,* in January 1806, to draw up a succinct account of his opinion on the cause and cure of this malady. The indefatigable attention to the avocations of his profession, the extent of his practical knowledge, and, above all, his disposition to feel no influence but that of duty, fully justified the choice of the Admiralty. There were two sources, Dr. Baird believed, in which the ship-ulcer originated: want of cleanliness in the ship, and negligent or improper management on the part of the surgeon. It is obvious that these causes may act separately; but the probability is, that when the Captain becomes careless of the state of the vessel, the Surgeon will be inattentive or slovenly in the discharge of his professional duty. In dirty ships, (a comprehensive term, including the actual impurities that may be suffered to accumulate in every part, the want of ventilation, and inattention of the crew to personal cleanliness) this disease was found most to prevail. Neglect on the part of the Surgeon is specifically stated to be inattention to slight hurts; not enjoining rest to patients before extensive inflammation, the precursor of gangrene, took place; and using the same sponge and water indiscriminately to all cases of ulcer. Under these circumstances it was, that blistered parts, punctured veins, recent wounds, and abraded surfaces, degenerated into malignant ulcer. The endeavour to rescue the navy from this calamity, was necessarily cessarily divided into two branches: the *prophylaxis and therapeutica.* The first was intimately connected with the discipline of the ship, and not only became a means of prevention, but an able auxiliary in the cure. The detail of this comprehended a general attention to cleanliness; an enlargement of the sick, and an erection of a convalescent berth for the reception of all recent wounds and clean sores; and into which, all cases that had gone thro' sphacelation, and had assumed a healthy appearance (the patient's person and cloathing being previously purified) were removed. No sponge, or any kind of dressing used in the foul ulcer ward, was suffered to approach the convalescent berth. Of the particular nature and quality of this ulcer, Dr. Baird expressed himself with a force and precision, that could only arise from extensive observation, and a conviction that his opinions were founded on truth. He contemplated it as a disease of increased action, and requiring a treatment in its early stage similar to other inflammatory affections; and calculated to render less destructive its subsequent progress. The medical practitioner who is employed in those departments of civil life least liable to be influenced by impure air and sordid living, frequently encounters peculiar idiosyncracies, in popular but expressive language, called *bad habits of body*, where the smallest wound, or abrasion of surface, is followed by active inflammation, ulceration, and sometimes gangrene. In these cases, without being influenced by the prospect of future sphacelus to employ stimulants and tonics, the judicious practitioner looks only to the controul of the increased action, knowing that the ease or difficulty of the subsequent management will be proportioned to the extent of the inflammation. It seems that this peculiar idiosyncracy, or bad habit of body, prevails very extensively among seamen, inducing upon all external injury, an active inflammation, to be restrained only by the most powerful remedies.

Upon this principle was founded the successful practice of Dr. Baird. In the first, or inflammatory stage, early rest was enjoined; the inflamed part was covered with compresses kept constantly wet with the vegeto-mineral water: the use of animal food and wine was strictly interdicted. Brisk purgatives were exhibited every second day, and saline draughts taken in the intervening one, with the addition of opium every five or six hours to allay irritability, and to procure sleep an anodyne always at night. As soon as the inflammation flammation was arrested, simple dressings were applied; and if the disease ran into sphacelus, an employment of the fermenting cataplasm assisted in producing an early separation of destroyed parts. When the sphacelated parts were separated, the same simple dressing was again used, or adhesive straps if the bone was sound. By a moderate use of animal food, wine and bark, the strength was gradually restored. The propriety of this mode of treatment is established on its uniform success: "for ulcers are now, where these doctrines are acted upon; almost extinguished from the service." The once rapid spread of this ulcer through the fleets, and into the naval hospitals, seems principally to have been induced by a false theory. When a contagious or malignant property was only looked to, stimulant and tonic remedies were exclusively employed internally, and irritating antiseptics applied to the ulceration. The inflammatory stage of the disease, and upon the conducting of which hung the final success, was either entirely overlooked, or its management so influenced by the idea of *malignity*, as to increase rather than abate its activity. Uninfluenced by hypothesis, Dr. *Baird* saw, that unless the inflammatory action could be kept within certain bounds, every remedial effort would be foiled: and it is to his good sense and practical skill that the country is indebted for the lessening, if not extinction, of this disease.

The work of M. Richerande, in 4 vols. having been published at Paris, 1808,* hardly comes into the plan of this *Sketch*: as it is, however, entirely practical, and professes, without any view to the historical progress of the art, to describe the scientific and operative part of modern Surgery, it becomes highly interesting to the English practitioner. The detail it gives of the state of surgery in France, affords the fair means of estimating whether in England or in that country, the science has made the greatest progress. But English surgeons will not subscribe to the decision of M. Richerande, when, on giving a comparative view of the art in different countries, he says,

"L'Eclat dont brillait la chirurgie Française devint pour le reste de l'Europe un utile sujet d'emulation. En ces tems vécurent en Angleterre Cheselden, Douglas, les deux Monro,

* Second edit. enlarged and improved,

Monro, Sharp, Cowper, Alanson, Pott, Hawkins, Smellie, et les deux Hunter; en Italie, Molinelli, Bertrandi, Moscati; en Hollande, Albinus, Deventer, Camper; en Allemagne, et dans le norde de l'Europe, Heister, Platner, Rœderer, Stein, Bilguer, Acrell, Callisen, Brambilla, Thedan, et Richter. Tous ces hommes célèbres s'accoutumènent à regarder l'Académie de Chirurgie comme le centre commun des lumières dont toutes les parties de l'art se trouvaient éclairées; la supériorité de la chirurgie Française fut généralement reconnue, et noblement avouée par la plupart d'entr'eux. Cet hommage ne lui fut pas rendu peut-être d'une manière unanime chez une nation rivale, qui, fière de ses Bacon, de ses Loke, et de ses Newton, aspire vainement a une supériorité trop universelle. Si les suffrages de l'Europe n'avoient pas fait justice de ces prétentions, et si d'ailleurs l'espace ne manquait à notre zèle, nous nous plairions, dans un parallèle des chirurgies Française et Anglaise, a comparer, et même à opposer Wiseman à Paré, Cheselden à J. L. Petit, Jean Hunter à Desault: dans cette espèce de lutte établie entre les chirurgiens des deux peuples, il n'est pas difficile de prévoir de quel côté resterait l'avantage."

The truth is, that each country has excelled the other in some particular points; and we sincerely regret that the evils of war prevent a participation of their peculiar improvements. If *M. Richerand* had been better acquainted with the state of English surgery, his work, as a view of the present state of the art, would have been more perfect; and his opinion of French surgeons might, perhaps, have come to a modester standard. On the operations for aneurism in particular, the French might receive much instruction. For, notwithstanding *M. Richerand's* opinion to the contrary, tying the external iliac, the carotid and subclavian arteries, may be effected without any extraordinary difficulty, and with great prospect of success. The year 1809 has produced cases of this kind. Mr. Abernethy, in his remarks on aneurism, attached to the "Observations, &c." relates three cases of tying the external iliac, two of which were completely successful; and Mr. Astley Cooper, in the Medico-Chirurgical Transactions, gives the histories of two cases of tying the carotid artery. A correct knowledge of the anatomy is too obvious to be mentioned as an indispensable qualification in the operator; but it seems of great importance to urge an early employment of the operation, and before the aneurismal tumour has injured, in any great degree, the surrounding parts; or become, from its extent and diseased state, the source of morbid irritations, which may themselves destroy the patient. While these bold operations receive their sanction from success, we feel it a duty to state, that the experience of this year seems to have fully determined, that one much more simple cannot be performed without putting life into extreme hazard. In certain varicose states of the *vena saphena*, it has been proposed to tie that vessel in a way that has some analogy to the operation for aneurism. This has several times been done, and the result has commonly been severe inflammation, which, in several instances, has ended in death.

The frequency of herniæ, the alarming symptoms that occasionally arise from them, as well as the hitherto unavailing efforts of art to discover any means of radical cure, must be an apology for noticing the following statement in a work entitled, "*Cochlioperie; Recuiel d'Experiences tres-curieuses, sur les Helices terrestres, ou Escarjot, &c. 8vo. Paris,* 1808." M. Tarenne asserts that he has discovered a radical cure for hernia. Having observed, he says, that the slimy juice of the snail had been long used with success in disorders of the breast, and noticing its astringency, and the singular reproductive quality which the animal itself possesses; he was led to conjecture that this juice, when applied upon the skin, would readily penetrate, and spread itself to the diseased parts beneath. Upon this principle he applied this agglutinating fluid to hernial apertures; and he further asserts, that he succeeded in radically curing the disease. Though there is no rational expectation that a cure can be thus effected, yet the positive manner in which M. Tarenne relates his cases, will induce some, possibly, to give his method a trial. To enable those who may be thus disposed, M. Tarenne's process is here inserted from a reputable periodical work. The common spring truss, when employed in this process, has its convex cushion made concave, for the purpose of receiving a china or glass cup, which cup is to have a diameter equal to the hernial aperture. This cup, intended to be kept upon the opening through which the intestine escapes, by the pressure of the elastic truss, is filled with wool imbued with the liquor flowing from the snail. Being provided with this apparatus, the patient is directed to proceed in the following manner: three or four hundred snails are to be procured in the spring, and kept in a place where they may have nutriment, as only from two to eight are to be used daily. The patient every day before he rises, and after he is in bed, bed, takes away the cup from the truss, and with a pin wounds the snail, at intervals, in different places. From each wound is given out through the opening in the shell, sometimes a blueish, sometimes a grey coloured water, which must be caught on the wool in the cup.* This being sufficiently filled with the liquor is to be placed on the part affected, always very exactly in the same situation; it is then to be covered with a white linen cloth, and the cushion of the truss applied over it. This treatment is to be pursued three or four months, changing the wool occasionally, and attending to other accidents, as inflammation or excoriation in the part where the cup is applied. By this method, says M. Tarenne, a common hernia may be cured in three or at least four months." It is evident that M. Tarenne assumes it to be a fact, that the fluid exuding from the wound made in the snail has an agglutinating property, or the faculty of producing something that will close the hernial aperture. We fear that this will not be found to be the fact, and that we shall have to regret, after many thousand of these little animals have been destroyed, that M. Tarenne's project will, for want of success, be forgotten. It has been the misfortune of the genus Helix to have affixed to it a vulgar opinion, of affording, in several of its species at least, a specific for some obdurate diseases. Even yet the *Helix pomatia*, found in Surry, particularly about Guildford, Dorking, Boxhill, &c. is sacrificed in incredible numbers, to make what the country people call *snail water*, a compound, possessed, in popular opinion, of extraordinary powers.

 The

* If it could be admitted that any kind of snail afforded a principle, or material, that had the property of reproducing destroyed animal substance, it would be highly desirable to ascertain that species: then would the vague description of M. Tarenne be regretted. Of the thirty-eight species of British Helices, we believe the *Helix hortensis* has been that most employed for medical purposes. Some disease of the chest, resembling *phthisis pulmonalis*, having got well during its use, has given it temporary fame; but its application to the true phthisis always destroyed that fame. Thus has it, from time to time, risen and fallen in public estimation. It is not, perhaps, so generally known that the garden-snail has been employed in ascites. In this disease, the bruised animal, in large quantity, made into cataplasm with barley flour, and a species of lichen not correctly described, is applied over the navel. In one instance, tolerably authenticated, and where the patient had filled after paracentesis, the application of this cataplasm was followed by complete recovery. The *Helix pomatia* is the most remarkable of the British species. This is principally, if not exclusively, found on the Surry Hills, where, in the month of May, the animal with its large

The theoretical and practical parts of medicine have received in the year 1809, considerable and interesting accessions. The attention that has been given to a train of diseases which have been considered as the opprobrium of the English climate, from the importance of their object, will claim the serious deliberation of the Faculty. There is some impregnation of the atmosphere of the British islands, or some impression from its vicissitudes, that render pulmonic complaints of more frequent recurrence among their inhabitants, than in other parts of the globe. These complaints assume a variety of forms, from the common catarrhal cough of the spring months, where the secreting membrane of the bronchiæ recovers from, or goes through the stages of the disease in ten or fourteen days, to chronic winter cough, serious pneumonic inflammation or bronchitis, and the destructive phthisis pulmonalis. All these depend, it is believed, on some state of the air; or, perhaps, on habits of life acting in conjunction with atmospherical vicissitudes except the phthisis, which possibly has its cause in some strumous state of the lungs, rather than in the atmosphere; though the accidents of that fluid will undoubtedly accelerate its progress, or bring into action the latent taint. Experience has often borne out the Brunonian doctrine of the cause of simple catarrh. The *Elementa Medicinæ,* agreeably to its leading principle, asserted that these affections of the bronchial membrane were occasioned by the sudden application of heat after exposure to cold. In the language of the system, this was explained to be an application of an exciting power, in a disproportionate degree to a part where the excitability, property, or susceptibility to be acted upon, was accumulated, from the absence or reduced standard of the exciting power. The consequence

---

large cream coloured shell, moving among the herbage, never fails to excite attention. The appearance of this snail in England is fixed to a precise period. Sir Kenelm Digby is asserted to have brought the first colony hither, with a view of employing the animal in medicine: and there is a tradition still existing at Dorking, that a person who had resided long in Spain, brought a colony to Box-Hill, for the purpose of employing them for soup, to the use of which he had been accustomed in that country. Several attempts have been made to rear the *Helix p.* in other parts of England, without effect; some have been brought to the gardens in the vicinity of the metropolis, but have soon died; indeed, it has been found impracticable to preserve them, even in the gardens of the town of Dorking; for they either escape from their new residence, or soon die in it. This snail was a favourite article of diet among the Romans, and Pliny speaks of its being enormously increased in size by feeding.

consequence of this would be, in the part where the excitability had accumulated from want of regular exhaustion, an increase of vascular action proceeding to that form of disease called inflammation. In this view of the cause of pulmonic complaints, it is evident that the hazard of taking cold, as it is popularly called, must be in proportion to the duration and degree of exposure to a low temperature, and to the degree and suddenness of the application of heat after this exposure. When heat is applied, under these circumstances, to the secreting surface of the bronchial membrane in a certain degree, common catarrh is produced: in another degree, severe, and often fatal, *bronchitis*. When the parenchyma of the lungs, or their investing membrane, feel the impression, pneumonia or pleuritis ensues. This doctrine receives elucidation and support, by recurring to the known effects produced on animal bodies by the disproportionate action of cold and heat. These effects are, on the surface of the body, pain, inflammation, and gangrene. Very slight attention to the subject will ascertain that the symptoms of a cold or catarrh, first appear in the evening when shut up in a warm room near to a fire, and while taking warm or hot fluids. And so distinctly may this sometimes be marked, as to shew that the affection appears first on that side of the face nearest to the fire. Whatever may eventually be the fate of the Brunonian theory, there exist a sufficient number of facts to prove the danger that results from the application of artificial heat, both in health and in disease. Possibly, the asserted increase of pulmonary complaints may arise from the great exposure to cold, the effect of modern cloathing opposed to close, carpeted, and heated rooms.' These observations apply principally to the prophylaxis; when disease has appeared, the object and the means will be different. The treatment of consumption, the most destructive of this morbid train, has been studied with an exactness that promised a successful result. But every method failing, the last resource is sought in a mild and unvarying temperature of air, at Lisbon, Montpellier, Nice, or Madeira. It has been thought that this steady temperature might be artificially produced in this country; and Dr. Buxton, in an "Essay on the Use of a Regulated Temperature in Winter Cough and Consumption," has pointed out its efficacy, and suggested a mode of execution. Something of this kind had been proposed a few years since by Dr. Adams, (Med. and Phys. Journ. v. 311.) and Dr. George Pearson, (Phil. Mag.) has lately projected a similar similar resource for those consumptive patients who cannot seek relief in warmer climates.

Fevers, especially the contagious and epidemic, have always been considered by the Faculty, as diseases of the most serious importance; and great labor has been employed in the investigation of their qualities, causes, and symptoms. The expedition to Walcheren has unfortunately afforded many opportunities of studying the epidemic of marshy countries; and probably much light will be thrown on the causes and peculiarities of this fatal disease. This year has, however, hardly allowed time to elucidate the character of this epidemic, or to ascertain the quality of the causes that produced it. In some seasons, the fenny parts of this kingdom have suffered under the influence of this disease, a devastation nearly equal to that which took place in the Walcheren expedition; and the progress of the fever has been as severe, allowing for the aggravation that all diseases experience from circumstances peculiar to the military life. It has never been discovered why the marsh remittent becomes epidemic in some years only. Within the memory of many of our readers, this fever has had an extraordinary prevalence in England. In 1780 and in 1808 this happened; but we have not learned that any peculiarity proper to these years has been detected, sufficient to explain the cause of this. The ingenious Inquiry into the Laws of Epidemics, by Dr. Adams, published this year, though it goes far in elucidating many circumstances belonging to this class of diseases, and makes nice discriminations between this cause of fever and contagions, does not very distinctly mark the character of epidemic seasons. The non-contagious nature of various fevers, which have generally been allowed to have a contagious origin, the plague in particular, he contends for with much curious research into their history, and much able reasoning; but which do not carry with them full conviction. The yellow fever, that source not only of destruction to human life, but of perpetual collision of opinion, we are again called upon to notice by Dr. Chisholm's Letter to Dr. Haygarth; in which he exhibits additional evidence of the infectious nature of this distemper, and endeavours to correct the pernicious, as he calls them, doctrines promulgated by Dr. Edward Miller and other American physicians, relative to this pestilence. Dr. Chisholm supports his opinion both by facts and reasoning, and with an earnestness that shews his own conviction. The American physicians have been extremely anxious anxious to convince their brethren in other parts of the world, that the yellow fever is not contagious, but is produced by the extrication of some gaseous effluvia from putrid vegetable matter. In 1807, this opinion was examined with as much attention as the limits of this Sketch permitted; and it was contrasted with the arguments for contagious origin. The reasonings on both sides were so equally poised, that the mind was left in a state of indecision. Neither does it appear that the physicians of America are perfectly decided in their opinion; for we have now to state the promulgation of a new hypothesis in that country, or rather the revival of an old one, to explain the origin, not only of *Typhus icteroides*, but of all epidemics.

Dr. John Crawford of Baltimore, in a Memoir entitled, "Remarks on Quarantines," published in a periodical paper called the Observer, endeavours by a long train of reasoning to prove, not only that the yellow fever, but all other febrile affections, *are consequences of animalcular action upon human bodies*. This, the wildest of philosophical vagaries, has taken full possession of Dr. Crawford, who, earnest in his opinion, is zealous for its support. He is, however, probably not aware of the progress of this hypothesis in Europe; and that very early, and at various subsequent periods, it has agitated the medical world. In the twelfth century, the Arabians had some loose theory of this sort; and in 1598, Dr. Mouffet, in the *Theatrum Insectorum*, mentions the circumstance.—But the earliest detailed and regular accounts of diseases of the most formidable nature being generated by animalcula, is in Hauptman's *Epistola Preliminaris Tractatui de viva Mortis imagine, &c.* published at Frankfort, 1650. Augustus Hauptman took the degree of Doctor in Physic at Leipsic, in 1653, resided at Dresden, and had the reputation of being an excellent chemist. He has been better known among physicians by his wild notions respecting the primary cause of diseases, all of which he regarded as arising from some species of vermes. The Treatise *de viva Mortis imagine*, the precursor to which was published in 1650, does not appear to have been printed; but the year after Hauptman took his Doctor's degree, he published, at Dresden, an 8vo volume, in which he treats of animalcula as the cause of diseases, with a minuteness and precision uncommon at that period. The *Scrutinum Pestis* of Kircher, published at Rome in 1658, supports Hauptman's opinions with much ingenious subtlety. Kircher was a man of deep learning, as is evinced by his *Œdipus Egyptiacus*

*Egyptiacus, Mundus subterraneus,* and the *Organon mathematicum.* He was also a bold and original thinker, more disposed to pursue his own opinions, than respect the sentiments of others. Though his writings abound with varied erudition, it is evident, that a warm imagination led him to seek the wonderful rather than the useful. With this diposition, at a period when the knowledge of nature was at a very low standard, he professed to explain the origin, cause, and prognosis of the plague, on the animalcular hypothesis. With these writers originated, we apprehend, the doctrine of the *materia contagiosa* being a living animalcule. In this hypothetical course, Hauptman and Kircher were soon followed by physicians of reputation for physiological learning, and a knowledge of nature.

Among the earliest of these is found Christian Langius, a French physician, born at *Luccau,* in the lower Lusace; and who, after quickly reaching the highest honours in his profession, died in 1662, at the early age of 42 years. In an introductory Preface to Kircher's *Scrutinum Pestis,* published at Leipsic in 1659, Langius probably first made public his opinions on the properties of Animalcula, as producing disease; but his doctrines were more precisely defined in a posthumous work entitled, *Miscellanea Medica Curiosa.* In this the *pathologia animata* is supported, not by fact and experiment, but by ingenious arguments drawn from the subtleties of scholastic reasoning, and conjectures founded on visionary hypotheses. England has not been without its supporters of this absurd doctrine. In a discourse on the state of health, in the island of Jamaica, published in 1679, the author, Dr. Thomas Trapham, attempts to prove that syphilis and yaws are caused by minute insects; and arrogates to himself the invention of the hypothesis. Dr. Bradley was an advocate for this doctrine, and derived his Animalcula from Tartary. Long after this, Dr. Adam Freer, in an inaugural Dissertation *de Syphilitide Venerea,* published at Edinburgh, supports a Theory so whimsical, that it is not easy to believe him in earnest. On the authority of Hauptman, Langius, and Bonomo, he assumes the opinion that itch and lues arise from different species of the genus *Acarus*; and that from the congress of the syphilitic and itch acari, a hybrid variety is produced, giving rise to sivvens. Strange as this may appear, a practitioner, who resided at Sandwich in Kent, and wrote a book with the quaint title of *Vermiculars examined,* went much further; for he undertakes to prove, prove, not only the existence of animalcules in all parts of the human frame, but that they are the cause of all diseases, and that every action of life originates with them. There has never been an opinion so absurd, but some theorist has adopted it with blind enthusiasm.

The ingenious surgeon, *Pierre Dessault*, of Bourdeaux, believed that syphilis was produced by animalcula, and that mercury proved a specific by the destruction it occasioned among this animated morbid material. In this opinion, like Trapham, he fails in originality; for it is evident, that his idea is taken from an hypothesis of *Antoine Dedier*, who resided at Montpellier in 1691, and died at Marseilles in 1746, when physician to the gallies in that port. In a dissertation *de Morbis Venereis*, published in 1723, *Dedier* argues, that an imperceptible vermis is the cause of syphilis; and that the disease is propagated by the transmission of this animalcule from one subject to another. But his hypothesis does not stop here. He warmly contends that the volatile and spirituous principle in vegetables depend on an assemblage of these worms. *Dedier*, notwithstanding his eager disposition to theorize, was a man of considerable learning, application, and ingenuity; of an open and ingenuous disposition, communicative of whatever he knew; and while physician to the *Hotel Dieu* at Montpellier, promptly answering all questions on his profession. It is probable his connection with *Raimond Vieussens*, whose daughter he married, and who was himself a wild and determined theorist, gave a bias to the mind of *Dedier*, fatal to his own reputation, and injurious to science.

As we approach nearer to the present times, and to an æra, when inquiries into the history of nature are not directed by the imagination, but by that sober faculty which admits nothing to be true that is not proved by carefully repeated experiments, we shall not find the animalcular hypothesis altogether abandoned. There was published at Vienna, in 1762, a volume, with the title of *Opera Medico Physica*, in which the author, *Maria Antonio Pleniz*, insists that bodies which are vulgarly supposed to rot, or to be dissolved by putrefaction, are devoured by myriads of animalcula; that the fœtor evolved from such bodies arises from the excrements of these animalcula; and that contagion is spread by their being wafted through the air, and carried from place to place. On this hypothesis, he attempts to account for the appearances in small-pox, measles, scarlatina, and all other contagious diseases.

The

The northern school of science, at the head of which was the immortal Linneus, has given a degree of consequence and credit to the animalcular hypothesis, which it did not, perhaps could not, receive from any other source. When this profound naturalist, in his *Exanthemata Viva* became the advocate of this doctrine, and supported it by observations scattered throughout his numerous writings, his pupils, who had been accustomed to look on his opinions as incontrovertible, labored to convince the scientific world, that this, the weakest of those opinions, was founded on the rock of truth.

In the *Exanthemata Viva,* published at Upsal, in 1757, Linneus admits many more insects as the cause of exanthematous diseases than he had seen; and thus, by reasoning from analogy, instead of supporting the theory by known facts, becomes as hypothetical as his predecessors. In an academical oration at Upsal, delivered with a florid rhetoric, perhaps pardonable on such an occasion, it is observed, that the Supreme Disposer of all Things has given command to those minute animalcules, the *Sirones,* and the whole man becomes one loathsome contagion. In the same oration, other species of *Sirones* are alluded to, and denominated the ministers of disease and death; the source of plague, small-pox, spotted fever, and other spreading and infectious disorders.

In the *Amenœtates Academicæ,* where the above oration is found, *Michael A. Baecknor* asserts, that dysentery, syphilis, and the contagions that produce exanthemata, are derived from some undiscovered species of *acari.*

The animalcular hypothesis, when employed to explain the contagions of exanthemata, dysentery, syphilis, and rabies; and to account for the paroxysms of epilepsy and the ravings of mania, rests solely on loose analogies and forced conjectures; and the admission of it generally, would have been fatal to the practice of medicine. Mercury, sulphur, and arsenic, would have been resorted to in all diseases, by physicians under the influence of this hypothesis; and medicines of the most pernicious qualities would have been given in pestis, variola, morbilli, scarlatina, &c. or possibly the principles laid down by a French empiric named *Boile,* might have superceded rational practice. About 1727, this person maintained at Paris, that all diseases were produced by animalcula in the blood, and different diseases by different animalcula; that there that there were other animalcula which were enemies to the noxious ones, and had the power to pursue and destroy them. He pretended to be well acquainted with the noxious animalcula, and with their several enemies, by means of which the sick might be relieved. He asserted also, that he well knew the medicines abounding with the auxiliary animalcules. All this he pretended to demonstrate by a microscope formed for the purpose; but the trick was soon discovered, and the Charlatan fled with his glasses and auxiliary animalcula.

In no other instance within the circle of theoretical speculation, has an hypothesis arisen so little capable of being subjected to practical purposes, as that which derives diseases from living animalcula. It is singular that this opinion, so long since rejected in Europe, should again be promulgated in America. But it is not by hypothesis, however cunningly supported, that improvement in the science of medicine is to be effected; on plain facts only can useful practice be founded.

From these visionary scenes we turn with increasing satisfaction to the sober path of scientific truth. Among the works that come nearest to this standard, we are induced particularly to notice the "Observations on some of the most frequent and important diseases of the heart; on aneurism of the thoracic aorta; on preternatural pulsation in the epigastric region; and on the unusual origin and distribution of some of the large arteries of the human body," by Mr. Allen Burns. A work intended to treat of all the diseases of the heart, should arrange them, in the opinion of Mr. Burns, under three distinct classes.

The first class should contain the sympathetic affections, or those diseases which are dependent on the consent which this organ has with other parts of the body.

The second class, those affections which are brought on by such malconformations of the heart, as give rise to a mixture of the venous with the arterial blood.

And the third, those diseases induced by some organic affection of the heart, which mechanically impairs the circulation, but not necessarily alters the composition or properties of the blood.

The second and third classes only are treated of in this work, in which many cases are inserted, and much practical information.

This addition to the history and treatment of the diseases of this important organ, will be properly placed in medical libraries libraries by the side of SENAC's admirable *Traite de la Structure du Cœur, de son action et de ses maladies.**

This year has produced further Observations upon two of the most dreadful maladies that afflict mankind, but without much prospect of lessening their fatality. Over cancer and hydrophobia, the medical art has hitherto maintained but little power; this should not, however, deter from investigation. In the cure of these diseases every thing is yet to be learned; their cause remains obscure, and the history of their characteristic symptoms is not sufficiently distinct. It would be desirable to see this investigation marked by attentive observation, by an unprejudiced examination of facts, and having the authority of sober experience instead of wild invention. Upon the first of these calamitous afflictions, *carcinoma,* and the peculiar tumour which is understood generally to precede it, Dr. Lambe has published "Reports," in which he details the effects of regimen. This physician has long cherished opinions upon the properties and powers of water, that mankind will not very soon be convinced are true. "Water is," he says, "a poison which affects every fibre of the body; it is the direct and immediate agent in the production of cancer, and may fairly be suspected to be operative in the production of all diseases attended with a solution of continuity." In a former work, to which this may be considered as the sequel, Dr. Lambe endeavoured to prove that all water contains arsenic; and that this is the material that gives to it destructive qualities. By distillation, he considers this fluid to be separated from its arsenical impregnation, and when thus purified, believes, by its constant use to the exclusion of every other potation, cancer may be cured; but he advises as auxiliary to this process a diet of vegetables in their raw state. Whether chronic and constitutional diseases may be mitigated or even cured, by a total change of all the ingesta, is not yet absolutely determined; and it is to be lamented, that Dr. Lambe's inquiry on this point has been generally hypothetical, and but little founded on a course of experiments.

* Tissot said of Senac's Work, "Cet ouvrage n'accroit rien laissé a desirer, si son illustre auteur, en annonçant une second edition, ne nous avoit pas appris, qu'il pouvoit le rendre encore plus parfait. Un grand homme peut se surpasser lui-meme, et voir un point de perfection que les autres ne desirent meme pas." Unfortunately, Senac did not live to produce this corrected and improved edition.

ments. On the subject of this disease, Mr. Carmichael has published a second edition of his Essay, enlarged by a great number of cases, in which his proposed remedy seems to have been of decided advantage. Mr. Carmichael's Essay contains many curious facts and observations on diseases, and on the effects produced upon them by medicine. One fact, in particular, states, that the carbonate of iron, when applied to simple ulceration, or venereal chancre, brings on violent pain and inflammation; but is completely unirritating to the cancerous sore. This fact has so frequently occurred to Mr. Carmichael, that he does not doubt its truth. If so, this substance affords, were it required, a criterion by which to distinguish cancerous ulceration.

The Medico-Chirurgical Transactions gives a very distinctly marked case of hydrophobia, by Dr. Marcet; which was likewise seen by Dr. Yelloly, Mr. Astley Cooper, and other Medical Gentlemen. It terminated fatally in five days. An examination of the body took place, which has not proved more explanatory than other dissections. There was some appearance of inflammation in the stomach; "the pharynx was considerably inflamed, behind both the mouth and nose; and in the œsophagus there were several detached spots of inflammation at various distances, in the course of the canal." Two practical inferences are deduced from the history of this case.

1st. It confirms the inutility of opium, and proves that the preparations of iron and of arsenic have no power over this disease.

2d. That as pain about the part bitten is one of the earliest indications of the virus becoming active, it shows, with much probability, that for an indefinite time, generally not less than three weeks, the poison remains at the part where it was inserted; and therefore it is never too late to employ the knife or caustic. As it cannot be proved that the virus spreads immediately from the part where it is inserted, or at all, until some local morbid action supervenes; no hypothesis should operate, at any period, to prevent the employment of excision, or other topical means for its destruction.

Dr. Geo. Lipscomb insists on the necessity of destroying the bitten part, either by excision or caustic, and recommends the employment of mercury; a medicine, which, notwithstanding its failure in every authentic instance, this writer still considers as fully equal to the cure of hydrophobia. If Dr. Lipscomb has experienced its efficacy in in so many instances, why are those instances concealed? Because the disclosure might expose the feelings of his patients, *says the Doctor*, to inquiries highly "indecorous and distressing," and to "curiosity, indelicate or impertinent." We consider it as a great misfortune, that the delicate feelings of Dr. Lipscomb's patients should prevent the publication of that evidence which alone can establish his assertions. But if the opinion and conclusions of Mr. Maryan are to be admitted, society will sustain no loss by this suppression of evidence. It has been the fate of *rabies* to excite, at one time, the most serious alarm; and at another, to have its very existence, as a disease *sui generis*, denied. After a detail of cases, and a series of observations so recently reported, and authenticated by men of undoubted veracity and knowledge, some surprise was occasioned by a Treatise "explaining the impossibility of the disease termed hydrophobia being caused by the bite of any rabid animal." The reasonings of Mr. Maryan will no more induce the public to suffer dogs afflicted with this disease to range without controul, wherever their disorder impels, than the arguments of those ingenious gentlemen, who would persuade the world that plague is not contagious, will make the governors of states neglect every means to prevent its importation.

Neither can we yet be persuaded, that the hypothesis of Mr. Maryan, or the assertion of Mr. Lipscomb, in favour of a particular remedy, will prevent the honest and intelligent practitioner pursuing the only right mode of inquiry. By a close, faithful, and unprejudiced observation of symptoms throughout every stage of the disease; by an attention to the apparent effects of medicine upon it, making an accurate distinction between those effects and its natural phenomena; and by a minute examination of morbid appearances on dissection, by men duly qualified, much may yet be done, to combat this hitherto invariably fatal disorder.*

There

* The inhabitants of *Hoya de Castalla*, in the Southern part of Valencia, have, from time immemorial, been in possession of a remedy for the bite of the viper. This remedy is composed of sea holly, (*Eryngium maritimum*), vipers bugloss (*Ecnium vulgare*), thorny madwort, (*Alyssum spinosum*), and cretan balm (*Melissa cretica*).† These plants are taken when

† The identity of the *Melissa cretica* seems doubtful. Cavanilles says, the plant used is the *Nepeta marifolia*. We are still as much at a loss. What is the *Nepeta Marifolia*?

There is another point connected with this inquiry, which, if properly attended to, may further elucidate the natural and medical history of rabies. The progress of disease among dogs and other animals presents a wide and almost uncultivated field for investigation. By exploring this, discoveries may possibly be made of essential importance to the right understanding of hydrophobia. In France, the disease has been propagated among quadrupeds by inoculation. In the preceding numbers of this Journal, Mr. Surr has given an interesting, and we trust a faithful history of this disorder, as it appeared in horses; and Dr. Jenner, (Medico-Chir. Trans. vol. I. 1809) by Observations on the *Distemper* in Dogs, has set an example highly worthy of imitation; and has accurately taught us to distinguish between that disease and *Rabies.* The distemper has the marked character of being "as contagious as the small-pox, measles, and scarlet fever, among the human species; and the contagious miasmata, like those arising from the diseases just mentioned, retain their infectious properties a long time after separation from the distempered animal." From the study that has been applied to the diseases of quadrupeds, since veterinary medicine has become a branch of regular scientific education; and, more especially, from the attention that has been given to the *variola vaccinæ,* from the time Dr. Jenner brought it before the public, it may be expected that a full and accurate view

---

when they begin to run to seed, and dried in the shade till all their humidity is evaporated. Each is then separately pulverized, and passed through a hair sieve. These powders are mixed together in equal quantities, to form the medicine. The dose for a man, 1 scrup.; for a dog, 1 dr.; to be taken morning and evening, for nine days, in wine and water. We have been induced to copy this recipe from the positive assertion that the Spanish Botanist, *Cavanilles,* as well as some physicians of Spain, have found it a completely successful remedy for hydrophobia.

*marifolia?* The *Nepeta cataria,* a native of Britain, possesses some active qualities, and should not be overlooked in this inquiry. It is tolerably well authenticated that this plant occasions abortion in cattle that chance to feed on it; and cats are so delighted with it, that they cannot be kept out of the garden where it grows. Ray asserts, that cats will not touch the plants raised from seeds, but only the transplanted ones: and Millar confirms this from his own experience. This fact was probably first noticed from an old dogrell rhyme.

*If you set it,*
*The cats will eat it;*
*If you sow it,*
*The cats will not know it.*

The undeniably powerful effect the *N. cataria* has on the nerves of the cat, may encourage a slender hope; where every thing has hitherto failed, it should be tried. The Journal from which the Spanish recipe is copied, blunders so much on the Botanical names, calling Sea Holly *Eryngium campestre,* that it cannot claim much confidence; and perhaps, *Nepeta marifolia* is of its own creation.

view will soon be had of the morbid derangements of this class of animals.*

We have already seen a disease of the cow, which a few years ago was either unknown or unnoticed, exciting the observation of mankind in all parts of the world; and leading to physiological and pathological disquisitions that develope some mysteries of animal life, in a manner, at once interesting to the philosopher, and beneficial to society. In the progress of the inquiry into those diseases among quadrupeds, which are connected with, and influence the health of the human race, it is to be much regretted, that so small a portion of liberality has been exercised. Indecorous language and finesse have marked the conduct of some busy supporters of vaccination, in this country at least; while its opposers have invented unsuccessful cases, distorted truth, and abused the public feeling, by fabricating relations of new and loathsome diseases, arising from the introduction of this morbid poison into the human frame. Though the disgraceful manner in which this controversy has been conducted subsides, as the persons originally concerned in it retire from the combat; yet vaccination itself is, perhaps, in more danger from the scientific scrutiny to which it has this year been subjected. Indeed, from attacks conducted with moderation, liberality, and science, it can only be defended by the truth of its principles, and the efficacy of its practice. Among many publications on vaccination in 1809, the most considerable are Thomson's plain statement of facts in favour of cow-pox. Smith's comparative advantages of cow-pox and small-pox inoculations. Brice's practical observations on the inoculation of cow-pox. Brown's inquiry into the anti-variolous power of vaccination. Report of the surgeons of the Edinburgh vaccine institution; containing an examination of the opinions and statements of Mr. Brown. Brown's letter in reply to this report. And Carneiro's reflections and observations on the practice of vaccine inoculation.

The last in this list, purporting to be the production of a Portuguese physician, is remarkable for its prejudices, blunders, and false statements. Hostile to the cause of vaccination,

* A Treatise on the Diseases and Management of Sheep, by Sir G. S. Mackenzie, affords a valuable accession, this year, to veterinary medicine; and gives the best detail hitherto made public, of the treatment of the disorders incident to this useful animal, illustrated by its Anatomy.

vaccination, it repeats the absurdities of the lowest English anti-vaccinists; and from the circumstance of its promulgation among a superstitious and ignorant people, it is formed to produce an effect injurious to a cause which every friend to science and to mankind must wish to see fairly investigated.*

The practical part of medical literature has been further increased by Mr. Ward's facts, establishing the efficacy of opiate frictions in spasmodic and febrile diseases; Observations on the use of mercury in hepatic affections, by Drs. Saunders and Curry; by a republication of Professor Hamilton's treatise on female complaints; and by a third edition of Dr. James Hamilton's observations on the utility and administration of purgative medicines in several diseases. Also by a new edition of Mr. Hunter's treatise on the venereal disease, with a commentary by Dr. Adams. Dr. Arnold's observations on the management of the insane; and Cheyne's pathology of the larynx and bronchia. The *Ars Obstetrica* and the *Materia Medica* have likewise received considerable additions.

In the first, Mr. Burns's principles of Midwifery; and in the second, a new edition of the Pharmacopœia of the College of Physicians of London, claim particular attention. Notwithstanding we already possess some valuable systematic treatises on the principles and practice of midwifery, the work of Mr. Burns cannot be considered as superfluous. It comprehends the practical improvements of the present period, and gives a full and faithful view of the science as it now appears. In particular, it has the excellence of connecting every subject, directly or indirectly appertaining to the theory or practice of obstetrics. After a lapse of twenty years and upwards, the additions made to the catalogue of our *Materia Medica*, or a more correct knowledge of the properties of substances already admitted into that catalogue; an improved nomenclature of chemistry, and a profounder acquaintance with the principles and practice of that art; the general prevalence of terms employed in the sciences of botany and natural history, were deemed by the College of Physicians of suf-

* With much satisfaction we have learned that the test of re-inoculation with variolous virus has been employed in Dublin, to a considerable extent, on the vaccinated of various periods; and without, in any instance, producing small-pox. This fact is authenticated by the signatures of many respectable surgeons in Dublin.

ficient force to induce a republication of their Pharmacopœia, in a form corresponding with, and adapted to, the present improved state of scientific knowledge. Two translations of this work have appeared, one by Dr. Powel, and the other by Dr. Campbell. A practical *Materia Medica*, a *Pharmacopœia Chirurgica*, and Dr. Thornton's elaborate Herbal, containing the properties of plants used in medicine, diet, and the arts, are to be added to this enumeration. The authority from whence the London Pharmacopœia proceeds, gives to it an elevation of station, which equally displays its excellencies and its defects. No human production is faultless. Whether the London Pharmacopœia has a larger proportion of this penalty of man than should in reason belong to it, party will determine; but with a spirit that will hardly admit a cool and candid judgment. Errors certainly it has; but these seem more the fault of inattention than of intellect. Of the two translations, it may be said, that they have mistakes which might and ought to have been avoided. Upon very different ground are they, however, censurable. Dr. Powell owes to his station, and to the College, more attention and care than his translation exhibits. Dr. Campbell, when one of his objects, at least, in publishing, was to reprehend Dr. Powell, should himself have been free from errors equally glaring, and more dangerous.

A mode of relieving, and sometimes of curing diseases, of very antient application, and when subjected to proper management, of extraordinary efficacy, has, this year, had the chance of being elucidated by the taste and liberality of a gentleman who possesses all the means for the encouragement of science that ample wealth affords.

The honourable Basil Cochrane, from observations on the *vapour bath* in India, and other places, and perhaps excited by the publication of Dr. Kentish's Essay on warm and vapour baths, has erected, at his house in Portman-square, an ingenious and extensive apparatus, which, by various mechanical contrivances, controuls the vapour of boiling water in a manner that renders it of easy application. The construction of this apparatus and its application, Mr. Cochrane communicates to the public in a 4to. volume, with eleven explanatory plates, under the title of " An improvement in the mode of administering the Vapour Bath, and in the apparatus connected with it; with the plans of fixed and portable baths for hospitals and private houses, and some practical suggestions on the efficacy of vapour, in application to various diseases of the human frame, and as may be beneficial to the branch of veterinary ty medicine." It has been the fate of the *vapour bath* to have often been the resort of empiricism; and the positive assertions of projectors and partizans, that it has suddenly cured the most obstinate diseases, have had the misfortune to give it an air of charlatanism, which it has not always deserved. Under the impression of this fact, our anxiety to guard against the designs of quackery, whether it attacks from batteries disguised by the humanity of the honourable and the affluent, lurks in the ambuscade of diurnal paragraphs, or impudently issues to the destruction of mankind in the armour of a patent, may occasion observations that appear to implicate Mr. Cochraue. To obviate this impression, it becomes a duty to say, that a fairness and honourable candour are manifest in the views of this gentleman, accompanied with a desire to lessen the sufferings of human nature. Though we ad mit, in the fullest manner, the liberality of Mr. Cochrane's intentions, we see no reason to change an opinion matured by many years of reflection, that the powerful and rich will afford more effectual means for the improvement of medical science, by an encouragement of persons practically employed in its cultivation, than by attempting themselves to cure diseases; or by fancying that the *therapeutica* can be suddenly improved by means adopted on slight evidence, founded on single facts often ill understood, and by hastily-drawn inferences. The most sanguine can hardly expect that the experience and observation of Mr. Cochrane can add much to medical science; but if he has enabled those who are in the constant habit of watching the progress of disease, to observe the effects produced by the application of heat in the form of vapour, he has benefited society more essentially than he can by any detail of morbid affections managed by himself. When we hazard these observations, we do not mean to detract from the liberal ingenuity of Mr. Cochrane; but we know there is a habit, which from long exercise becomes almost intuition, that enables the medical practitioner to observe, and distinguish with acuteness and precission, the smallest shades of deviation from the healthy state; and to discover with quick penetration the nature and quality of symptoms, extremely obscure, doubtful, or absolutely unintelligible to the gentleman doctor. It is this habit too which enables the physician to discriminate the causes of cures, and which are often very different from those which popular opinion assigns. Under the direction of the gentleman whose name appears to certify the practicable ingenuity of the construction of these baths, the curative powers of the vapour of heated water may be fully ascertained; and we anxiously hope that this may not prove another ephemeral project, but that the enthusiasm of Mr. Cochrane, under the guidance of men of science, and learned in the operations of nature, not only by study but by habit, may prove what the vapour bath will, and what it will not do.

The sciences considered as auxiliary to the medical art have not been neglected; and if their career has not been so brilliant in this as in some former years, still that career is not without interest. Botany, Chemistry, and Natural History, have each had some additions in 1809. Mr. Joseph Knight has published some ingenious and practical observations on the cultivation of the plants belonging to the natural order of *Proteceæ,* with their generic and specific characters, and places where they grow wild. A second edition of Dr. Smith's valuable introduction to physiological and systematic botany, has appeared; and a new edition of a very pleasing little book, with the title of Elements of the Science of Botany, as established by Linnæus, with examples to illustrate the classes and orders of his system.

Chemistry, which, by the genius of Mr. Davy, has been raised in England to a very exalted station, still pursues its investigations with alacrity. The researches of this chemist, directed to the decomposition of bodies hitherto thought elementary, are employed with an ardour that probably will effect discoveries of great importance.

The French chemists, since Mr. Davy's brilliant discovery of the base of alkalies, have been deeply engaged on that subject; and have been able to procure *potassium* by chemical affinities, without the aid of galvanism. The inquiry into the constituent principles of bodies by chemical analysis, has been followed with much assiduity; and when employed with a view of developing their properties and powers, has been of much service to society. On this subject, the chemists of France have done much; neither have those of England been idle. Dr. Bostock, as noticed in the preceding part of this Report, has been engaged in examining the constituent parts of the blood; and has also instituted an experimental inquiry to ascertain the real nature of vegetable astringents. M. M. Vauquelin and Le Sage have analysed the juice of the *atropa belladonna,* and made experiments with it on animals. M. Nyston has injected various gases into the blood vessels of animals, and reports reports the effects produced on those animals, and the changes occasioned in the blood itself.

A subject of great interest to the farmer, and not very remotely connected with the practice of physic, has employed the talents of M. M. Fourcroy and Vauquelin. They have analysed the smut of wheat, which is stated to consist of phosphoric acid, phosphoric salts, a carbonaceous matter, and a fœtid oil; and is gluten in a state of decomposition. There is no circumstance that has excited in the agriculturist a greater degree of anxious curiosity than this peculiarity in wheat. Many hypotheses have been formed as to its nature and origin: sometimes it has been asserted to originate with animalculæ, or to be animalculæ itself: at another, it is a parasitical fungus, a non-descript agaric: and now it is a gluten under particular circumstances. Perhaps even yet we do not understand its real nature and properties. The farmer believes it to have a contagious principle, and is eager to free the mass of sound grain from it by washing with water, with solutions of sea salt, arsenic, or sometimes an alkaline ley. Secret, and if we mistake not, patent nostrums have been employed in England to remove or eradicate this animal or vegetable contamination from among sound wheat. Various opinions have been entertained of the efficacy of the means employed; but it seems, as the object is to remove the particles of this substance merely, that a strong solution of *potass* promises to succeed best. This singular substance, whatever it is, has a peculiar fœtor, well known to farmers and millers; and which it imparts, with its dark colour, to meal.

Persons compelled to eat bread made of meal thus contaminated, may, it is not improbable, have their health much injured. And in this point, it becomes to the physician an important object of inquiry. That those singular cases of gangrene which have been attributed to feeding upon meal made from injured corn, or from corn having a large admixture of *lolium temulentum,* were not occasioned by smut, may be, at least, doubted.

The remark that was applied to Richerand's work on Surgery, is also suitable to the ingenious Memoir of the celebrated traveller Humboldt, "*sur l'Anguille electrique du Nouveau Continent,*" which was published at Paris, about the same time with the *Nosographie Chirurgicale.* If the connection of natural history with the medical art is more remote than botany and chemistry, still in many instances it strongly illustrates the physiological branch of that art; and, as in the present, may possibly tend to explain some

 very very obscure functions of the animal machine. In this Memoir of M. Humboldt, will be found extremely curious facts respecting the *gymnotics electricuns*. The method of taking the fish, the history of its powers, with observations on those circumstances, afford not only an entertaining relation, but materials for prosecuting the history of Animal Electricity. Perhaps the circumstance of its instantly losing its electric power on the separation or destruction of the brain, may lead to further knowledge of the *modus operandi* of that organ and the nerves.

The importance of Medical Jurisprudence we have always felt it a duty to assert, and the still doubtful Liverpool Case, again occasions it to be noticed. The controversy arising from that case has extended to the present year; and two pamphlets, on opposite sides, shew distinctly how far it has excited a local sensation. Mr. Dawson's "Exposure of some of the false Statements contained in Dr. Carson's Pamphlet," and Dr. Campbell's "Reflections on the Vindication of the Opinions delivered by the medical witnesses for the Crown," will probably terminate the dispute: but not with it, we trust, the impression which the subject and the mode of investigating it has produced. About the period of the trial at Lancaster, another remarkable one, for procuring abortion, took place at St. Edmunds Bury. A curious physiological fact was subjected to some investigation at this trial. It became an object to the Court to ascertain the period of quickening,* and whether the fœtus then first receives the principle of animation. The evidence on this question, shews how vaguely professional men understand the subject; and how indeterminate the law upon it is, from a want of precise knowledge of the fact. The parties escaped punishment in this instance, because they employed the effectual mode of procuring abortion, when the law only reaches a method that is always ineffectual.

The

* Quickening, as it is popularly termed, is a sensation of a peculiar quality, occurring to most women at some period of utero-gestation, (between the 10th and 25th week, says a writer of great authority;) and is believed to be the first indication of life in the fœtus. Perhaps this sensation has no dependance on the life of the fœtus, but is simply produced by the impregnated uterus rising suddenly out of the pelvis into the cavity of the abdomen. This peculiar sensation occurs but once; and is never reproduced by any subsequent motions of the fœtus. A very little attention might ascertain the fact. Do women labouring under a retroverted uterus ever experience the

The benefit that has arisen from establishing literary and scientific societies, has not operated to a greater extent in any branch of human learning, than in the medical art. In 1808, we endeavoured to point out the advantages of such institutions, though in a manner confessedly inadequate to describe their extensive utility. Our attention is again called to the subject by the Medical and Chirurgical Society of London having published the first volume of its Transactions. In the year 1805, several physicians and surgeons of the metropolis of the British Empire, "held a meeting for the purpose of *considering the best means of obviating the acknowledged want of a Society founded upon liberal and independent principles, and conducted with propriety and dignity.*" The result of this meeting was the formation of the *Medical and Chirurgical Society of London,* composed certainly of a most respectable portion of the medical talent and consequence of the metropolis Several papers in the volume published by this Society, have already been noticed; the remainder are of a practical nature generally, and are indebted for their utility more to the quantum of their facts, than to the plausibility of their theories.

The writer of this *Annual Historical Sketch of the Progress of the Medical Art,* has occasionally added his mite of information to the subjects that came under his notice, by such remarks and short details as the subjects themelves suggested; and especially while giving a history of passing events, has he believed it proper to strongly impress the value of veracity, and to inculcate an unbiased study of Nature. "THE VOLUME OF NATURE IS THE BOOK OF KNOWLEDGE; AND HE BECOMES MOST WISE WHO MAKES THE MOST JUDICIOUS SELECTION."

*Princes Street, Cavendish Sqaare,*
*June* 15, 1810.

---

the sensation of quickening? If this explanation of a singular fact in the female economy should hereafter be ascertained to be false; still, as our present uncertainty on the subject gives occasion to much physical or metaphysical jargon; and especially as the laws recognize the circumstance to mark the line of division between the legal innocence and criminality of certain actions, it is not undeserving inquiry.

